# Seminal vesicle status and its association with semen parameters in congenital bilateral absence of the vas deferens (CBAVD)

**DOI:** 10.1186/s12610-025-00267-0

**Published:** 2025-06-18

**Authors:** Iman Shamohammadi, Abdolreza Haghpanah, Ali Eslahi, Mohammad Ali Sadighi Gilani, Ali Adib, Seyyed Sadra Kashfi

**Affiliations:** 1 Department of Urology, School of Medicine, Shiraz University of Medical Sciences, Shiraz, Iran; 2 Department of Urology, School of Medicine, Shariati Hospital, Tehran University of Medical Sciences, Tehran, Iran; 3 Urology and Nephrology Research Center (UNRC), Center of Excellence in Urology, Shahid Labbafinejad Hospital, Shahid Beheshti University of Medical Sciences, Tehran, Iran

**Keywords:** CBAVD, Vas deferens, Seminal vesicle, Infertility, Absence bilatérale du Canal déférent, Vésicule séminale, Rein, Infertilité

## Abstract

**Background:**

Congenital bilateral absence of the vas deferens (CBAVD) is a rare condition associated with male infertility. CBAVD is often accompanied by other genitourinary anomalies, including abnormalities or agenesis of the seminal vesicles and kidneys. While it is expected that CBAVD always coexists with seminal vesicle agenesis due to a shared embryologic origin, some studies report the presence of seminal vesicles in certain CBAVD patients. This study aims to assess the status of seminal vesicles in CBAVD patients and explore the relationship between seminal vesicle presence and semen parameters.

**Methods:**

In this multi-center, retrospective cross-sectional study, we reviewed data from 47 CBAVD patients diagnosed between 1994 and 2024. Data collected included demographic information (age, height, weight, BMI), physical examination findings (vas palpation), and imaging results (trans-rectal ultrasound for seminal vesicle status, abdominal and pelvic ultrasound for kidney status). Laboratory data included serum FSH, LH, and testosterone levels, as well as semen analysis results (pH, fructose, and volume). Patients were classified into three groups based on seminal vesicle status: bilateral agenesis, unilateral agenesis, and bilateral presence.

**Results:**

Among the 47 CBAVD patients, 29 had bilateral agenesis of the seminal vesicles, 9 had unilateral agenesis, and 9 had bilateral presence. No significant differences were found between the groups regarding weight, height, BMI, or serum levels of LH, FSH, and testosterone. Additionally, semen analysis revealed that 89.4% of patients had abnormal pH, 93.6% had abnormal volume, and all patients had abnormal semen fructose. There were no significant differences between the groups in semen pH, fructose, or volume.

**Conclusion:**

Our findings suggest that the presence or absence of seminal vesicles in CBAVD patients does not significantly affect semen parameters. This may be due to dysfunction of the seminal vesicles in those with a present organ.

## Résumé

**Contexte:**

L’absence bilatérale congénitale du canal déférent (CBAVD) est une maladie rare associée à l’infertilité masculine. La CBAVD s’accompagne souvent d’autres anomalies génito-urinaires, notamment des anomalies ou une agénésie des vésicules séminales, et des reins. Bien que l’on s’attende à ce que la CBAVD coexiste toujours avec l’agénésie des vésicules séminales en raison d’une origine embryologique partagée, certaines études rapportent leur présence chez certains patients atteints de CBAVD. Cette étude vise à évaluer l’état des vésicules séminales chez les patients atteints de CBAVD et à explorer la relation entre présence de vésicules séminales et paramètres du sperme.

**Résultats:**

Dans cette étude transversale rétrospective multicentrique, nous avons examiné les données de 47 patients atteints de CBAVD diagnostiqués entre 1994 et 2024. Les données comprenaient des informations démographiques (âge, taille, poids), les résultats de l’examen physique (palpation du canal) et les résultats d’imagerie (échographie transrectale pour l’état des vésicules séminales, échographie abdominale et pelvienne pour l’état rénal). Les données de laboratoire comprenaient les taux sériques de FSH, LH et testostérone, ainsi que les résultats d’analyse du sperme. Les patients ont été classés en trois groupes en fonction de l’état de la vésicule séminale: agénésie bilatérale, agénésie unilatérale et présence bilatérale.

Parmi les 47 patients atteints de CBAVD, 29 présentaient une agénésie bilatérale des vésicules séminales, 9 une agénésie unilatérale et 9 une présence bilatérale. Aucune différence significative n’a été observée entre les groupes en ce qui concerne le poids, la taille, l’IMC ou les taux sériques de LH, de FSH et de testostérone. L’analyse du sperme a révélé que 89% des patients avaient un pH anormal, 94% un volume anormal, et tous les patients avaient un fructose anormal. Absence de différences significatives entre les groupes en ce qui concerne le pH du sperme, le fructose ou le volume.

**Conclusions:**

Nos résultats suggèrent que la présence ou l’absence de vésicules séminales chez les patients atteints de CBAVD n’affecte pas de manière significative les paramètres du sperme. Cela peut être dû à un dysfonctionnement des vésicules séminales chez les personnes qui ont cet organe présent.

## Introduction

Congenital bilateral absence of the vas deferens (CBAVD) is a rare anomaly and is the cause of around 1–2% of infertility cases [1]. Most patients are first diagnosed due to infertility and their evaluations demonstrate azoospermia because of the absence of both vas deferens. The prevalence of the vas deferens absence is supposed to be about 0.1%, but because patients with unilateral forms of this disease may be asymptomatic fertile men, it is underestimated [2]. Although CBAVD is often a manifestation of cystic fibrosis (CF), it can be isolated [3, 4].

In addition to infertility, CBAVD may present with other symptoms. CBAVD is often accompanied with other genitourinary anomalies including abnormalities or agenesis of the seminal vesicles and kidneys [5–7]. It is expected that CBAVD is associated with seminal vesicle agenesis in all patients due to their common embryologic origin. The epididymal duct, the vas deferens with the ampulla, the ejaculatory duct, and the seminal vesicles all originate from the wolffian ducts [3]. However, there are studies that report some CBAVD patients with present seminal vesicles [8–10]. Jarvi et al. indicated that abnormality in the distal wolffian duct structures, such as the ampulla of the vas deferens and seminal vesicles, is dependent on the CFTR genotype as half of the patients with CBAVD who do not carry any CFTR mutations present with bilateral presence of seminal vesicles [11].

Overall, bilateral seminal vesicle (SV) anomalies are observed approximately twice as frequently in individuals with congenital bilateral absence of the vas deferens (CBAVD) compared to those with congenital unilateral absence of the vas deferens (CUAVD), with rates of 50% versus 25%, respectively. In contrast, unilateral SV anomalies are predominantly found in cases of CUAVD (80%), where they are typically ipsilateral (9).

When CBAVD is associated with bilateral absence of seminal vesicles, this triad will be detected in semen analysis as well as azoospermia: low semen volume (< 1.5 ml), acid pH (< 7.0), and low seminal fluid fructose (< 13 μmol/ejaculation) [3]. However, low semen volume may be due to obstruction of the ejaculatory duct in such patients with present seminal vesicles and CBAVD.

Physical examination of the scrotum for palpating the vas deferens cannot be reliable for all patients, especially those with residual vas deferens or fibrous cord-like structures remaining after vas deferens atresia or those with obesity or with high-riding scrotums [9, 12]. Hence, ultrasonographic imaging should be performed in the evaluation of these patients [13].

Despite common belief, which considers infertility in CBAVD patients as a result of the absence of the vas deferens and consequently prevention of sperm release, Cai and Li reported that these patients’ testes could produce sperm [9]. Therefore, these individuals seem to have offspring through ART methods [14–16].

In this study, we aimed to determine the status of seminal vesicles in CBAVD patients and assess the association between the presence of seminal vesicles and semen parameters.

## Methods

This study was approved by the ethics committee of Shiraz University of Medical Sciences (IR.SUMS.MED.REC.1403.586). In a multi-center retrospective cross-sectional study, from 1994 to 2024, the patients who had been diagnosed with CBAVD in Royan infertility clinic, Tehran, Iran, and Shiraz infertility clinic, Faghihi hospital, Shiraz, Iran, were enrolled. A total of 61 patients were evaluated. Of these, 14 were excluded from the study due to incomplete file information. Finally, 47 patients were included, and their profiles were reviewed for data extraction.

**Fig. 1:**
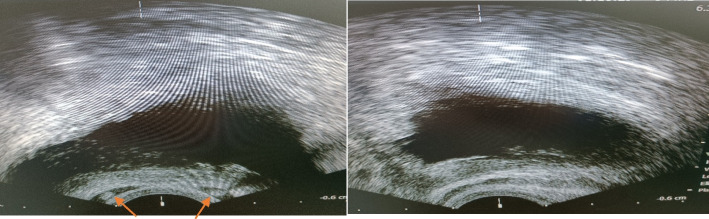
TRUS images of two CBAVD patients. The left side shows bilateral presence of SV indicated by arrows, while the right side shows bilateral absence of SV

All patients were examined by a single urologist. All these patients also underwent trans-rectal ultrasound and abdominal and pelvic ultrasound by a single radiologist. Data including age, height, weight, body mass index, vas palpation in physical examination, seminal vesicles feature based on trans-rectal ultrasound (TRUS), is shown in Fig. 1, and kidney status based on abdominal and pelvic ultrasound were extracted. The kidney status of each patient was considered normal, ectopic, or agenesis.

Laboratory data included serum follicle stimulating hormone (FSH), luteinizing hormone (LH), and testosterone level. All hormonal analyses were conducted at 8 a.m. Additionally, semen PH, fructose level, and volume were obtained from semen analysis reports, collected after an average abstinence period of 72 h. Semen PH less than 7.2, fructose level less than 120 mg/dl, and volume less than 1.5 cc were considered abnormal.

Patients were classified into three groups based on the presence of seminal vesicles, including bilateral agenesis, unilateral agenesis, and bilateral presence.

The collected data were analyzed using SPSS software version 26. Continuous variables were reported as means, and categorical variables were shown as frequencies. Continuous variables were compared using One-way ANOVA test, and categorical variables were compared using Fisher’s exact test. P-values ​​less than 0.05 were considered statistically significant.

## Results

The mean age of all patients was 33.14 ± 5.43 years, and the difference between the mean age of the three groups was not significant (Table 1). There were 29 patients with bilateral agenesis of SV, 9 with unilateral agenesis of SV, and 9 with bilateral presence of SV. Demographic information and hormonal assays of the three groups are shown in Table 1. There was no significant difference between them regarding their weight, height, BMI, serum LH, FSH, and Testosterone (Table 1).

**Table 1:** Demographic information and hormonal assays of the patients

Variables	Total (*n* = 47)	Unilateral SV agenesis (*n* = 9)	Bilateral SV agenesis (*n* = 29)	Bilateral SV present (*n* = 9)	P- ^a^value
Age, year; mean ± SD	33.14 ± 5.43	36.51 ± 6.14	32.96 ± 5.00	30.44 ± 4.95	0.06
Weight, kg; mean ± SD	83.56 ± 15.30	85.00 ± 10.65	82.90 ± 16.00	84.33 ± 18.18	0.63
Height, cm; mean ± SD	172.08 ± 11.50	173.44 ± 4.85	170.87 ± 13.97	174.80 ± 5.43	0.93
BMI, kg/m2; mean ± SD	28.81 ± 9.73	28.24 ± 3.10	29.40 ± 11.93	27.50 ± 5.30	0.87
LH, mIU/mL; mean ± SD	4.94 ± 2.37	5.22 ± 2.24	4.41 ± 1.53	6.84 ± 4.22	0.07
FSH, mIU/mL; mean ± SD	5.00 ± 3.44	5.10 ± 3.84	5.20 ± 3.60	4.03 ± 2.77	0.76
Testosterone, nmol/L; mean ± SD	5.14 ± 3.40	4.62 ± 2.40	4.82 ± 3.67	7.12 ± 2.63	0.30

^a^using One-way ANOVA test

Semen parameters are demonstrated in Table 2. Of the total 47 CBAVD patients, 89.4% had abnormal PH, 93.6% had abnormal volume, and all of them had abnormal semen fructose. There was no significant difference between the three groups regarding semen PH, fructose, and volume (Table 2).

**Table 2:** Semen parameters of the patients

**Variables**	**PH**		**P value**	**Fructose**		**P value**	**Volume**		**P- **^c^** value**
	** > 7.2**	** ≤ 7.2**		** > 120**	** ≤ 120**		** > 1.5**	** ≤ 1.5**	
Total	5(10.6)	42(89.4)	0.99	0.0	47(100)	0.99	3(6.4)	44(93.6)	0.62
Unilateral SV ^b^ agenesis; N (%)	1(11.1)	8(88.9)		0.0	9(100)		1(11.1)	8(88.9)	
Bilateral SV agenesis; N (%)	3(10.3)	26(89.7)		0.0	29(100)		2(6.9)	27(93.1)	
Bilateral SV present; N (%)	1(11.1)	8(88.9)		0.0	9(100)		0.0	9(100)	

^b^seminal vesicle

^c^using Fisher’s exact test

Of the total 47 CBAVD patients, four patients (8.5%) had ectopic kidney, and three patients (6.3%) had kidney agenesis (Table 3). All patients with ectopic kidneys had bilateral SV agenesis, and two patients with kidney agenesis presented with unilateral SV agenesis, which was ipsilateral to the kidney agenesis in both cases. We did not find any abnormality in the kidneys in patients with bilateral presence of SV. However, none of these patterns had a significant difference with the condition of SV.

**Table 3:** Kidney status of the patients

Variables	**Total**	**Unilateral SV agenesis**	**Bilateral SV agenesis**	**Bilateral SV present**	**P- **^d^** value**
Normal kidney; N (%)	40(85.1)	7(14.9)	24(51.1)	9(19.1)	0.53
Ectopic kidney; N (%)	4(8.51)	0.0	4(8.51)	0.0	
Agenesis kidney; N (%)	3(6.38)	2(4.25)	1(2.13)	0.0	

^d^using Fisher’s exact test

## Discussion

In this study, we evaluated 47 patients with CBAVD regarding the presence or absence of SV. Of them, 61.8% had bilateral absent SV, 19.1% had bilateral SV, and 19.1% had unilateral SV. Hormonal assays of our patients revealed normal LH and FSH alongside decreased testosterone levels, consistent with previous studies [16–18]. However, no significant difference was observed among the three groups in the presence or absence of SV in our study.

Because of a similar embryologic origin, it is expected that SV is absent in CBAVD patients. However, several studies have revealed that patients with CBAVD can have unilateral or bilateral SV [8–11, 19–21]. The existence of SV in these patients may be due to the normal caudal portion of the mesonephric duct, despite its abnormal rostral portion [9]. However, it seems that these organs are dysfunctional in CBAVD patients, even with normal appearance. The results of our study confirmed this issue. We did not find any difference between the semen parameters related to SV functions in CBAVD patients with and without SV. Therefore, the results of semen analysis in patients with CBAVD can be reliable. While some CBAVD patients may present with normal semen parameters such as fructose, pH, and volume, these cases warrant thorough clinical evaluation to rule out misdiagnosis or coexisting anatomical abnormalities, such as Zinner Syndrome, which involves SV cysts and ipsilateral renal agenesis [22].

Strong evidence for diagnosis of CBAVD relies on imaging modalities. In such patients with residual vas deferens or fibrous cord-like structures remaining after vas deferens atresia, the diagnosis of CBAVD may be missed [12, 23].

A case report described a patient with CBAVD without SV anomalies [9]. The vas deferens were palpable bilaterally. Semen analysis showed normal volume, normal fructose levels, and azoospermia. TRUS confirmed the presence of bilateral seminal vesicles. Scrotal exploration revealed aplastic vas deferens with a blind-ending tail on the left and absence on the right. Vaso-epididymal anastomosis was performed due to suspected seminal tract obstruction. This case was notable for CBAVD with normal semen fructose and volume.

However, among 47 patients of our study, only three had normal semen volume, and interestingly, none of these patients had bilateral seminal vesicles. There was no significant difference in semen parameters between patients with and without SV who had CBAVD. Our findings were in the same line with those of the study by Taille et al., in which semen variables (pH < 7.2, fructose < 1 g/L, and ejaculate volume < 2 mL) did not differentiate patients with or without SV anomalies [24]. So, it can be concluded that the presence of SV with normal appearance in patients with CBAVD does not necessarily indicate functional competence. Regardless of their presence or absence, SVs are unable to contribute effectively to seminal fluid production due to underlying dysfunction, whether or not a functional epithelium is present.

Another study evaluated 26 men with CBAVD with computerized tomographic scans [8]. They found seminal vesicles bilaterally in 12 of these men, unilateral hypoplasia or absence in 8, and bilateral hypoplasia or absence in 6 subjects. They failed to find reconstructable vas deferens when exploring the patients with bilateral or unilateral present SV. Thus, they concluded that low ejaculate volume and absence of semen fructose in men with CBAVD may be related to ejaculatory duct problems rather than SV. They did not find significant differences among semen parameters between these men, like our results, but we mentioned that abnormality in semen parameters in patients with CBAVD that had normal SV might be related to dysfunction of SV rather than ejaculatory duct anomalies.

In our study, 40 patients had normal kidneys, four had ectopic kidney, and three had kidney agenesis. Although our results demonstrated that all kidney abnormalities were observed in patients with SV abnormalities, we found no significant association between kidney anomalies and the presence or absence of SV. Results of a study conducted on 26 cases of CBAVD demonstrated that four patients had unilateral kidney agenesis, and one patient had a hypoplastic pelvic kidney. All four men with unilateral kidney agenesis had ipsilateral absence of the SV [8]. Another study showed that among 168 cases of CBAVD, 17 men had unilateral kidney agenesis. However, no significant difference was observed in the seminal vesicle status between the group with both kidneys and the group with a single kidney [21]. It seems that kidney anomalies cannot be predicted in CBAVD patients with SV agenesis.

To the best of our knowledge, this study is one of the few studies that have evaluated the relationship between the presence or absence of SV with semen parameters and kidney abnormalities in CBAVD patients.

Small sample size is an important limitation of our study. Because of the retrospective design of our study, we had to exclude some patients with missing profile data. Also, one of the major limitations of this study was the absence of genetic variant analysis of the CFTR gene mutations. Due to the prohibitively high costs, genetic analysis of CFTR mutations has performed only on the spouses of the study cases. As all results were negative, genetic variant screening for these genes was not conducted in the study participants.

Additionally, the seminal vesicle volume, if present, was not measured, which highlights the need for further research in this area.

## Conclusion

Based on our findings, the presence or absence of SV in CBAVD does not significantly affect semen parameters. This is because, even when SV are present, they do not effectively contribute essential seminal fluid components, regardless of epithelial functionality.

Further studies to evaluate the seminal vesicles and the causes of their dysfunction in these patients are recommended.

## Data Availability

The datasets used and/or analyzed during the current study are available from the corresponding author upon reasonable request.

## Abbreviations

CBAVD: Congenital bilateral absence of the vas deferens
CF: Cystic fibrosis
SV: Seminal vesicle
CUAVD: Congenital unilateral absence of the vas deferens
TRUS: Trans-rectal ultrasound
FSH: Follicle stimulating hormone
LH: Luteinizing hormone
CFTR: Cystic fibrosis transmembrane conductance regulator
